# Nephro-Zebra: acute kidney injury secondary to rhabdomyolysis—a rare and reversible cause

**DOI:** 10.1007/s40620-021-01166-z

**Published:** 2021-11-01

**Authors:** Samuel A. Watson, Kerry-Lee Rosenberg, Kate Bramham, Helen Alston

**Affiliations:** King’s Renal Unit, King’s College Hospital, London, SE5 9RS UK

## Case description

A 65-year-old male patient presented to a routine renal outpatient appointment. He had been successfully treated for kappa light chain myeloma two years previously, during which time he developed a severe acute kidney injury (AKI) requiring haemodialysis. Renal function had recovered over a period of 4 months, leaving him with a new baseline creatinine (Cr) of 190–200 µmol/L (chronic kidney disease (CKD) stage 3B)s.

The patient reported a clinical history of insidious-onset fatigue and new orthopnoea and reduced exercise tolerance over the preceding 3 weeks. Clinical examination revealed a 17.8 kg weight gain (79.0–96.8 kg), with associated peripheral and periorbital oedema. Renal function tests demonstrated a new AKI (Cr 289 µmol/L), as well as hypocalcaemia (corrected Ca 2.12 mmol/L) and hypomagnesaemia (0.44 mmol/L).

Treatment with oral furosemide 80 mg daily and oral calcium and magnesium supplementation was commenced. The patient was discharged with advice to represent if his symptoms worsened. He was reviewed in the renal ambulatory unit 7 days later. Improvement of presenting symptoms was reported and substantial diuresis had been achieved, with a reduction in body weight to 88.8 kg and improvement of oedema. However, the patient reported a new symptom of muscle cramping. Repeat blood tests revealed a worsening AKI (Cr 361 µmol/L), grossly elevated creatine kinase (6322 IU/L) and thyroid function tests in keeping with hypothyroidism (thyroid stimulating hormone (TSH) > 100m IU/L, free thyroxine < 1.3 pmol/L).

The patient was admitted for further investigation.

*What is the cause of this patient’s acute kidney injury, fluid overload and rhabdomyolysis? What are the diagnostic tests required to establish a diagnosis?*

## Case solution

Several differential diagnoses were considered. Renal autoimmune tests, urinary tract ultrasound scan and renal artery/vein duplex scan were normal. Urine protein/creatinine ratio was mildly elevated (29.9). Serum free light chains were stable and there were no concerns for a myeloma relapse from the Haematology team. Cardiac causes were ruled out with normal NT-proBNP (< 20 pg/mL) and trans-thoracic echocardiogram. Mild derangement of liver function tests (aspartate transaminase 139 IU/L, gamma-glutamyl transferase 67 IU/L) and a normocytic anaemia were found (Hb 127 g/L). Review of the patient’s regular medication did not reveal any associated with rhabdomyolysis.

Thyroid autoantibodies were requested given the presence of hypothyroidism. Anti-thyroglobulin (799 U/ml) and anti-thyroid peroxidase (TPO—274 U/ml) antibody titres were raised. A diagnosis of rhabdomyolysis-induced AKI, secondary to a first presentation of Hashimoto’s thyroiditis was made.

*How does hypothyroidism induce rhabdomyolysis and subsequent AKI?*

Hashimoto’s thyroiditis is the most commonly identified cause of hypothyroidism. Anti-thyroglobulin and anti-TPO antibodies produce an autoimmune inflammatory reaction within the thyroid, resulting in hypothyroidism. Myopathy affects approximately a third of patients with hypothyroidism, with the most frequent symptoms reported as weakness and muscle cramps. The development of rhabdomyolysis, however, is rare and the mechanism has not been fully elucidated. Hypotheses include impaired mitochondrial oxidation and decreased myocyte carnitine levels, the development of an insulin resistant state, decreased protein turnover and direct autoimmune mechanisms [1, 2]. The development of renal impairment due to rhabdomyolysis can be caused by multiple mechanisms. These include renal vasoconstriction and subsequent ischaemia, intraluminal obstruction by myoglobulin and uric acid cylinders, and direct ferrihemate toxicity [3].

*Can hypothyroidism result in fluid overload via other mechanisms?*

Impaired water excretion due to both direct and systemic effects is another recognised complication of hypothyroidism. Due to effects on the cardiovascular system, a lack of free thyroxine results in reduced cardiac output and blood pressure, ultimately resulting in reduced renal blood flow. Human radioisotope studies have demonstrated this corresponds with a rise in creatinine due to actual changes in glomerular filtration rate. Preclinical studies have demonstrated that a lack of free thyroxine can result in both dysregulation of aquaporin I and II expression and a reduced ability to achieve maximum urine dilution due to non-osmotic vasopressin release [4].

### Treatment and follow up

**Fig. 1:**
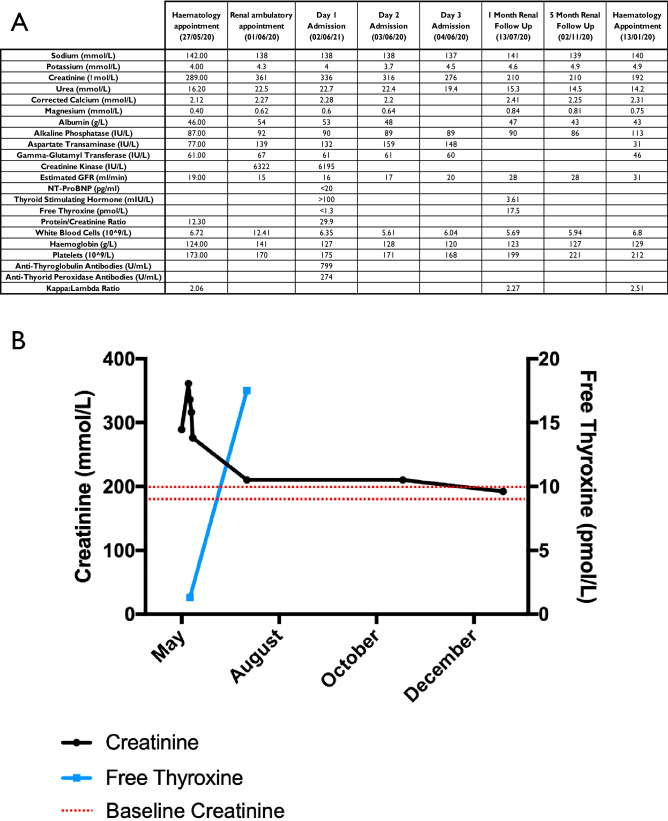
**A** A selection of relevant blood tests relating to this case report. Units for each parameter can be found in brackets in the left-hand column. **B** Visual representation of Creatinine & free thyroxine levels over time

The patient was started on 100 µg oral thyroxine daily and discharged. On review 1 month later, creatinine had returned to near baseline (Cr 210 µmol/L) and both TSH (3.61 IU/L) and free thyroxine (17.5 pmol/L) were within normal limits. The patient reported full resolution of symptoms and there was no clinical evidence of fluid overload. Renal function returned to baseline by 7 months (Cr 192 µmol/L)—all data presented in Fig. 1. Return to baseline renal function following initiation of thyroxine replacement after a first presentation of rhabdomyolysis-induced AKI secondary to a first presentation of hypothyroidism has only been described once previously [5].

## Conclusions

Hypothyroidism is an important differential diagnosis in rhabdomyolysis-induced acute kidney injury. The rapid initiation of thyroxine replacement can reverse kidney injury and prevent chronic renal impairment.
